# Measuring electronic health literacy in the context of diabetes care: psychometric evaluation of a Persian version of the condition-specific eHealth literacy scale for diabetes

**DOI:** 10.1186/s12911-024-02594-0

**Published:** 2024-07-05

**Authors:** Maryam Peimani, Mozhgan Tanhapour, Fatemeh Bandarian, Ensieh Nasli-Esfahani, Afshin Ostovar

**Affiliations:** 1 Diabetes Research Center, Endocrinology and Metabolism Clinical Sciences Institute, Tehran University of Medical Sciences, No. 10, Al-e-Ahmad and Chamran Highway Intersection, Tehran, 1411713136 Iran; 2 Health Information Technology Department, School of Allied Medical Sciences, Urmia Medical Sciences University, Urmia, Iran; 3 Metabolomics and Genomics Research Center, Endocrinology and Metabolism Molecular-Cellular Sciences Institute, Tehran University of Medical Sciences, Tehran, Iran; 4 Endocrinology and Metabolism Research Center, Endocrinology and Metabolism Clinical Sciences Institute, Tehran University of Medical Sciences, Tehran, Iran

**Keywords:** eHealth literacy, Type 2 diabetes mellitus, Reliability, Validity, Cultural adaptation, Scale

## Abstract

**Background:**

The rise of the internet and social media has led to increased interest among diabetes patients in using technology for information gathering and disease management. However, adequate eHealth literacy is crucial for protecting patients from unreliable diabetes-related information online.

**Objective:**

To examine the psychometric characteristics and explore the preliminary validity of the Persian version of the Condition-specific eHealth Literacy Scale for Diabetes (Persian CeHLS-D) to assess eHealth literacy in the context of diabetes care.

**Methods:**

After adapting, translating, examining content validity, and pilot testing the questionnaire, it was administered to 300 patients with type 2 diabetes mellitus (T2DM). Construct validity was assessed through confirmatory factor analysis, convergent and known-groups validity. The internal consistency (Cronbach’s alpha), composite reliability and maximum reliability, and test-retest correlation were assessed.

**Results:**

Factor analysis supported the hypothesized two-factor model with 10 items, and the standardized factor loadings ranged from 0.44 to 0.86 (*P*-values < 0.001). Cronbach’s alpha and test-retest correlation were good for each factor. Convergent validity was confirmed by significant correlations of Persian CeHLS-D with diabetes health literacy, perceived usefulness and importance of using the internet for health information, internet anxiety, and perceived physical and mental health. Know-groups validity determined using groups with different internet-use frequencies, and different attitudes towards providing online healthcare services, were satisfied.

**Conclusion:**

This study demonstrated the Persian CeHLS-D as a reliable and valid measure of eHealth literacy among patients with T2DM in Iran. Its satisfactory psychometric properties support its use in research and clinical settings to assess eHealth literacy and inform interventions.

**Supplementary Information:**

The online version contains supplementary material available at 10.1186/s12911-024-02594-0.

## Introduction

Type 2 diabetes mellitus (T2DM) is a chronic metabolic disease characterized by hyperglycemia and complex self-management tasks [1]. Patients with T2DM require high-quality, evidence-based information to facilitate informed decision-making. Compared to patients with other chronic diseases, such as cardiovascular and respiratory diseases, patients with T2DM show a greater need for information to manage their disease effectively and prevent complications [2].

This constant need for information can be overwhelming for patients with diabetes, especially those who are not familiar with the healthcare system. In recent years, with development of internet and social media, many patients have shown an interest in using technology for obtaining information and managing their diabetes [3, 4]. In a recent study, around half of the participants used the internet to search for self-management information, with dietary planning being the most frequent use [5]. The internet provides ubiquitous access to a vast repository of information from diverse sources. This information can be retrieved from any location in the world, at any time, with an internet connection. This has significantly accelerated and simplified the process of information retrieval, compared to the past, when individuals relied on libraries, encyclopedias, and other physical sources of information [6, 7].

Due to such dynamic changes in the information acquisition landscape, there is an undeniable need to ask about the competencies of diabetes patients who are looking for information about their health online. These competencies were defined as eHealth literacy. The world Health Organization (WHO) defines eHealth literacy as the ability to find, understand, appraise, and use digital health information and services to inform and support health decisions [8]. For example, searching information about diabetes treatment options requires identifying appropriate and reliable sources and assessing quality of information by patients [9]. In addition, many web-based interventions have been designed and developed for diabetes self-management and patient empowerment [7, 10]. Therefore, healthcare providers should assess their patients’ eHealth literacy levels before providing them with technology-based education or interventions.

Although several generic instruments have been developed over the last two decades to assess eHealth literacy in a broad general population [11], there are also some disease-specific eHealth literacy skills that are important for people with specific diseases and should be considered in the instruments’ content. These skills are the skills and knowledge that people with specific diseases need to find, understand, appraise, and use digital health information and services to manage their condition effectively. For example, patients with T2DM need to be able to understand and use blood glucose monitoring apps and online food diaries, understand specific medical terms related to their disease, and figure out numeric medical examination values such as HbA1C values. Moreover, patients with different diseases have different searching interests. Diabetes patients are more likely to search for information on their medications compared to a healthy population [12, 13]. Hence, to better assess eHealth literacy among patients with diabetes, it is recommended that the instrument’s content be adjusted to reflect diabetes-specific conditions and concerns [14].

To date, there have been very few condition-specific instruments that measure eHealth literacy in a specific disease, such as the Transactional eHealth Literacy Instrument, developed for older adults with chronic lung disease [15]. Recently, one instrument has been developed specifically for patients with diabetes, designated as the Condition-specific eHealth Literacy Scale for Diabetes (CeHLS-D) [16]. The development of this instrument took place in South Korea and has exhibited good psychometric properties of construct validity and internal consistency in Korean patients with T2DM. However, more research is needed to evaluate its test-retest reliability and cross-cultural validity [16]. Therefore, the present study aimed to provide a comprehensive psychometric evaluation of a Persian version of the CeHLS-D in Iran.

## Materials and methods

### Study design

This cross-sectional psychometric evaluation study was conducted in two phases: (1) developing a Persian version from the CeHLS-D (translating, reviewing, and pilot testing), and (2) field testing and psychometric evaluation. Data collection for phase 1 was completed from December 2022 to March 2023 and for phase 2 from April 2023 to July 2023.

### Ethical considerations

The study protocol was reviewed and approved by the medical research ethics committee of the Tehran University of Medical Sciences (IR.TUMS.EMRI.REC.1401.093). Participants were given written and verbal information about the study, including what it was for, what it would involve, and that they could choose to stop taking part at any time. By answering the questionnaire, participants agreed to take part in the study. Participants were also told that their information would be kept secret and stored securely. STROBE guidelines were used to ensure the reporting of this study.

### Phase one: developing the persian CeHLS-D

In phase one, we explain the adaptation and translation of the Persian CeHLS-D, review by an expert panel for content validity, and pilot testing.

#### Adaptation and translation

CeHLS-D, recently developed (2021–2022) by a team of South Korean researchers, is a population-specific instrument applicable for type 2 diabetes in outpatient consultations [16]. It measures eHealth literacy specific to diabetes and its treatment and self-management in internet environments using digital devices. This instrument has a two-factor structure. The first factor measures a patient’s ability to find and understand diabetes health information online, and the second factor measures a patient’s ability to communicate with healthcare providers and other individuals online about their diabetes over the recall period as “at present”. CeHLS-D consists of ten items, each scored on a five-point Likert scale, with options ranging from 0 (“not at all”) to 4 (“very much”).

**Fig. 1:**
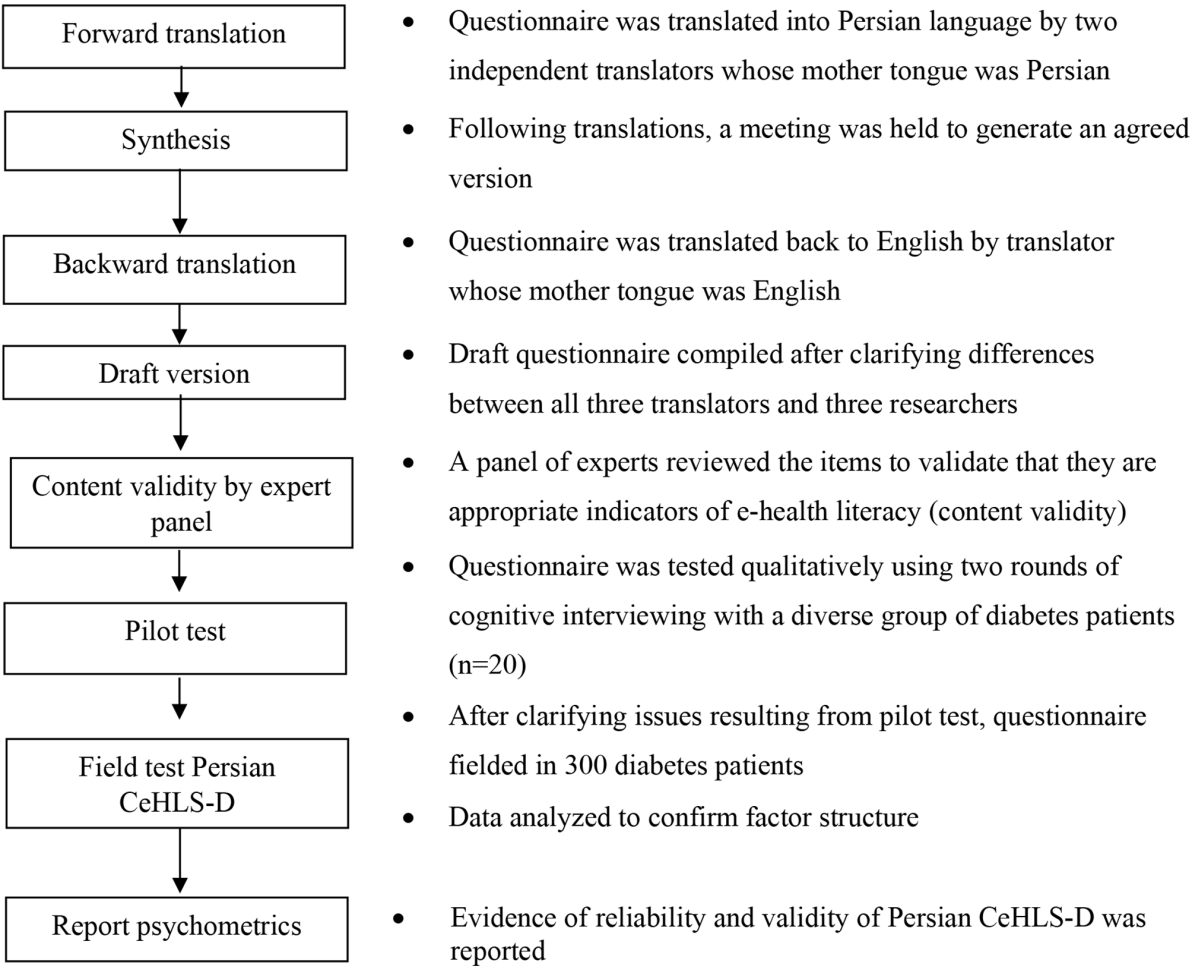
Overview of study methods

The cross-cultural validation adhered to WHO guidelines for the translation and adaptation of instruments [17]. The process involved forward translation, synthesis, back-translation, content validity by panel of experts, and pilot testing (cognitive interviewing with possible participants) (Fig. 1). Two independent translators translated the CeHLS-D into Persian. Both had Persian as the mother tongue, and were proficient in English. One was a professional translator with no particular knowledge about health literacy concept. The second was a nonprofessional translator, knowledgeable about health literacy. The translators met to agree on a single version (synthesis). Then, a third professional, bilingual, native English speaker back-translated the synthesized version to English. In a second meeting with all three translators and researchers, item by item, all versions and back translations were discussed to agree on an optimal version for semantic and conceptual equivalence between the English and Persian versions.

#### Content validity

To assess the content validity of the questionnaire, a panel of 10 experts with expertise in eHealth literacy, measurement properties, and diabetes care participated. They were asked to assess the necessity of each item based on their specialty on a 3-point Likert scale (1 = essential, 2 = useful but not essential, and 3 = not essential). These scores were then used to calculate a Content Validity Ratio (CVR) for the items. According to Lawshe [18], an acceptable CVR value for 10 experts is 0.62. The experts also rated the degree of relevance of all items to the overall concept of the scale on a 4-point Likert scale (1 = not relevant, 2 = somewhat relevant, 3 = relevant but needs revision, and 4 = completely relevant) to assess Content Validity Index (CVI. Scores of 0.79 or higher were considered acceptable for individual items [19].

The scale content validity index (S-CVI) and scale content validity ratio (S-CVR) were calculated by averaging the CVI and CVR values respectively. An S-CVI 0.9 and greater is considered acceptable [20].

#### Pilot testing

The resulting draft questionnaire was evaluated qualitatively using two rounds of cognitive interviewing with a diverse group of diabetes patients. Twenty patients were randomly recruited and interviewed after their visit in the diabetes specialty clinic. Each interview took approximately 90 minutes. We used elements of both the ‘think aloud’ and ‘probing’ approaches. In the first round, the patient read an item, and the interviewer asked if any words seemed difficult or confusing, and if the patient could describe how he/she understood the item. In the second round, we asked how the patient would interpret and respond to the items. Patients filled in the questionnaire item by item, sharing their thoughts about each item. After patients’ immediate thoughts about an item, the interviewer would ask how they understood the item.

### Phase two: field test of the persian CeHLS-D

#### Data collection, setting and participants

To field the questionnaire, a cross-sectional study was conducted in the diabetes specialty clinic affiliated to Tehran University of Medical Sciences in Tehran, Iran. Participants had to be at least 18 years of age with a diagnosis of type 2 diabetes, have access to electronic equipment with internet access, and have Persian as a native language. Sample size for the field test (*n* = 300) was calculated based on similar studies. Eligible patients were randomly approached by the researcher in the waiting room of the clinic and asked for permission to participate. Those who consented then completed a pen-and-paper survey in a private location in the clinic.

#### Measurements

*Diabetes Health Literacy.* The Persian version of Diabetes Health Literacy Scale (DHLS) was used to assess convergent validity [21]. The DHLS was developed to measure diabetes-specific health literacy, and comprises 14 items scored on a 5-point Likert scale from 1 to 5. The scale score is the average of all items, with higher scores indicating better health literacy. The Persian DHLS yielded excellent psychometric properties with high reliability and excellent convergence properties as well as factorial validity. Cronbach’s alpha of the scale in the present study was 0.90.

*Well-being.* The 5-item WHO Well-Being Index (WHO-5) was used to measure well-being. The WHO-5 consists of five questions with a 6-point Likert scale ranging from 0 (at no time) to 5 (all of the time). A higher score indicates a higher level of well-being. This scale can also be dichotomized for screening depression (a cutoff score of ≤ 50) [22].

*Internet use-related variables.* To assess the interest in using the internet, the following questions were asked: (1) How useful do you feel the internet is in helping you in making decisions about your health? (response options: not useful at all, not useful, unsure, very useful), (2) How important is it for you to be able to access health resources on the internet? (response options: not important at all, not important, unsure, important, very important [23].

One question was asked about the frequency of internet use: “How often do you use the internet to seek health information?” Response options were almost every day, several days a week, around one day a week, less than one day a week, and almost never [24].

Another question was also developed for this study with “yes, it has increased” or “no, nothing changed” as the response options: “Has the pandemic affected your use of the internet for health information?” We hypothesized that people with higher CeHLS-D scores (higher eHealth literacy skills) were more likely to report that the pandemic has increased their use of the internet for health information.

To assess the internet anxiety, the following three items were used: 1. The internet is something threatening to me, 2. I am afraid of making an irrevocable mistake while using the internet, 3. I am very concerned about the use of the internet. Response options rated on a 5-point Likert scale, ranging from 1 “does not apply at all” to 5 “applies completely” [25].

Furthermore, patients were asked about their level of agreement with providing the following healthcare services via the internet: consulting a physician, making an appointment online to see a physician, accessing medical records, accessing educational resources, accessing results of laboratory tests, and renewing prescriptions. Response options ranged from 5 (strongly agree) to 1 (strongly disagree). A higher score indicated a more positive attitude toward providing online healthcare services [26, 27].

*Sociodemographic and clinical characteristics.* Respondents provided demographic information including age, gender, level of education, marital status, occupation, and perceived health status. Data related to diabetes duration, treatment regimen type, diabetes complications, and HbA1c values were collected from electronic medical records.

#### Analysis of field test data

Statistical analyses were performed using SPSS version 24 and IBM Amos Graphics version 22. Descriptive statistics were provided for sociodemographic characteristics, clinical data, and internet use-related variables.

#### Validity

Construct validity was assessed through confirmatory factor analysis (CFA) and hypothesis testing (convergent validity and known-groups validity). Originally, the CeHLS-D scale was hypothesized as a two-factor model with seven items loading under “cognitive actions for internet diabetes information” and the next three items loading under “abilities of digital communication” [16].

Maximum likelihood estimation was chosen with output of squared multiple correlations, minimization history, standardized estimates, residual moments and modification indices. The model fit quality was assessed using the following indices: normed chi-square (χ^2^/df < 3), root mean square error of approximation (RMSEA) < 0.08, standardized root mean square residual (SRMR) < 0.08, Comparative Fit Index (CFI) > 0.90, and Goodness of Fit Index (GFI) > 0.90 [28, 29]. Moreover, the heterotrait-monotrait ratio of correlations (HTMT) was calculated to determine whether two factors were distinctly different from each other. An HTMT less than 0.85 suggested that the pair of factors was discriminant, meaning that they were sufficiently different from each other to be considered separate constructs [30].

We tested convergent validity by measuring the level of association between Persian CeHLS-D scores and diabetes health literacy. We expected significant positive associations with the diabetes health literacy score [16, 31], the importance and usefulness of using the internet for health information [32], health condition variables (perceived health status and well-being) [31, 33], and a negative association with internet anxiety [34]. The convergent validity between the Persian CeHLS-D and other constructs were assessed by computing Pearson correlation coefficients between continuous variables and Spearman correlation coefficients between ordinal variables. A correlation coefficient of 0.3 or less indicated a weak relationship, 0.3 to 0.6 indicated a moderate relationship, and 0.6 or higher indicated a strong relationship between the two variables.

We assessed known-groups validity by comparing the mean Persian CeHLS-D score in groups of people who use the internet more frequently than those who use it less. We expected people who used the internet more frequently would have higher eHealth literacy scores [31, 32]. We also compared the mean Persian CeHLS-D score in groups of participants who had a more positive attitude toward providing online healthcare services than those who had a less positive attitude. We expected that participants who had a more positive attitude toward providing online healthcare services would have higher eHealth literacy scores [27]. Known-groups validity was tested using a t-test or one-way analysis of variance (ANOVA).

#### Reliability

Construct reliability of the Persian CeHLS-D was assessed through internal consistency (Cronbach’s alpha), composite reliability (CR), and maximum reliability (MaxR) for each factor. It was assumed that Cronbach’s alpha, CR and MaxR should all be > 0.7 to indicate the reliability of the scale [35]. We examined item-to-total score correlations to determine if the item-to-total correlations were at least 0.30. To assess test/re-test reliability, 50 patients completed the Persian CeHLS-D two weeks apart and the Intra-class Correlation Coefficient (ICC) was calculated.

## Results

### Phase one: content validity and pilot testing

Based on expert recommendation, KakaoTalk, a Korean mobile text messenger, was replaced with Eitaa, a Persian language messenger, in item 9 of the CeHLS-D. This change was made to improve the cultural relevance and accuracy of the tool for the Persian population, as KakaoTalk is not used in Iran. All items met the criteria for content validity. The CVR for each item exceeded the recommended cutoff of 0.62, and all CVIs were 0.8 or higher. The S-CVR and S-CVI were calculated to be 0.85 and 0.92, respectively.

Results from cognitive interviewing in pilot testing indicated that all 20 patients were able to read, understand, and explain the items using their own words, confirming that the items were clear and easy to understand. A few minor problems were clarified through word revisions. For example, the item “ability to distinguish advertisements” was improved to “ability to tell the difference between ads and real information online” (Appendix 1).

### Phase two: characteristics of the study population in the field test

The mean age of participants was 61.11 years. Of the 300 participants, 156 (52.2%) were men, 129 (43.1%) had an education level of diploma (twelve years of education), 241 (80.2%) were married, and 123 (41.1%) were retired. The mean duration of diabetes was 11.69 years, and the mean HbA1C was 7.77%. More than half (52.5%) of diabetes patients indicated symptoms of depression (according to the WHO well-being index) and reported their health status was poor or fair (54.5%). Table 1 presents the sample characteristics of the study population in more detail.

**Table 1:** Participant characteristics (*n* = 300)

	Values
Age (years), mean (SD)	61.11 (9.01)
Gender, n (%)	
Male	156 (52)
Female	144 (48)
Educational level, n (%)	
Primary school	25 (8.3)
High school	37 (12.4)
Diploma	129 (43.1)
College/university (< 4years)	72 (23.8)
College/university (≥ 4years)	37 (12.4)
Occupation, n (%)	
Retired	123 (41.1)
Homemaker	95 (31.7)
Self-employed	54 (17.8)
Employee	22 (7.4)
Unemployed	6 (2)
Marital status, n (%)	
Married	241 (80.2)
Single	22 (7.4)
Widowed	27 (8.9)
Divorced	10 (3.5)
Perceived health status, n (%)	
Poor	64 (21.3)
Fair	100 (33.2)
Good	78 (26.2)
Very good, excellent	58 (19.3)
WHO well-being, n (%)	
Sufficient	142 (47.5)
Very low (≤ 50)	158 (52.5)
Diabetes duration (years), mean (SD)	11.69 (7.94)
HbA1C (%), mean (SD)	7.77 (1.75)
Treatment regimen, n (%)	
Oral anti-DM drugs	193 (64.4)
Oral drugs + Insulin	83 (27.7)
Insulin	24 (7.9)
Diabetes complications	
& comorbidities, n (%)	
Neuropathy	123 (41.1)
Retinopathy	46 (15.3)
Nephropathy	19 (6.4)
Diabetic foot ulcer	14 (4.5)
Cardiovascular diseases	85 (28.2)
Hypertension	150 (50)
Hyperlipidemia	224 (74.8)
Internet availability, n (%)	
Always available	126 (42.1)
Mostly available	83 (27.7)
Occasionally available	91 (30.2)
Not available	0 (0)
Frequency of internet use for health information, n (%)	
Almost every day	123 (41.1)
Several days a week	91 (30.2)
Around one day a week	36 (11.9)
Less than one day a week	33 (10.9)
Almost never	17 (5.9)
Has the pandemic affected the use of the internet for health information, n (%)	
Yes, it has increased	155 (51.5)
No, nothing changed	145 (48.5)
Internet Anxiety (Range 1–5), mean (SD)	1.97 (0.96)

### Psychometric analyses

#### General properties

The highest mean of the Persian CeHLS-D items was 2.45 for item nine, and the lowest mean was 1.27 for item eight. The mean scores for the total scale, factor 1, and factor 2 were 1.85 (SD = 0.79), 1.87 (SD = 0.77), and 1.78 (SD = 1.02), respectively (Table 2). Each of the 10 items were in the ± 2 range of skewness and kurtosis. None of the items showed a ceiling effect, but two items did exhibit a floor effect (items 8 and 10).

**Table 2:** Descriptive statistics of the Persian CeHLS-D items

Item	Mean (SD)	Skewness	Kurtosis	Ceiling Effect %	Floor Effect %	Item-total correlation	Intra-class correlation coefficient	Construct reliability
**Factor 1**: **Cognitive actions for internet diabetes information**	1.87 (0.77)	-0.12	-0.43	6	12		0.91	Cronbach’s alpha = 0.87 CR = 0.92 MaxR = 0.94
1. Thinking of search words	2.07 (1.04)	-0.203	-0.283	5.3	6	0.72		
2. Understanding medical terms	1.65 (0.90)	-0.078	-0.239	1	7.7	0.65		
3. Figuring out numeric medical examination values (e.g., HbA1c, fasting glucose)	2.12 (1.09)	-0.202	-0.344	7.3	6.6	0.59		
4. Appraising information credibility	1.71 (0.99)	0.169	0.064	3.7	8.3	0.83		
5. Distinguishing advertisements	1.96 (1.08)	-0.229	-0.553	4	8.3	0.72		
6. Trustworthiness of internet sources	1.65 (1.04)	0.039	-0.327	3	11.7	0.74		
7. Filtering applicable information	1.92 (1.03)	-0.283	-0.325	3	8.3	0.82		
**Factor 2**: **Abilities of digital communication**	1.78 (1.02)	0.22	-0.77	12.7	22		0.86	Cronbach’s alpha = 0.80 CR = 0.87 MaxR = 0.91
8. Emailing	1.27 (1.08)	0.698	-1.033	8.3	33.3	0.73		
9. Text messaging (e.g., WhatsApp, Eitaa)	2.45 (1.14)	-0.363	-0.471	14.3	4.7	0.72		
10. Sharing opinions on social media	1.63 (1.20)	0.151	-1.112	4.7	17.7	0.82		

#### Validity

**Fig. 2:**
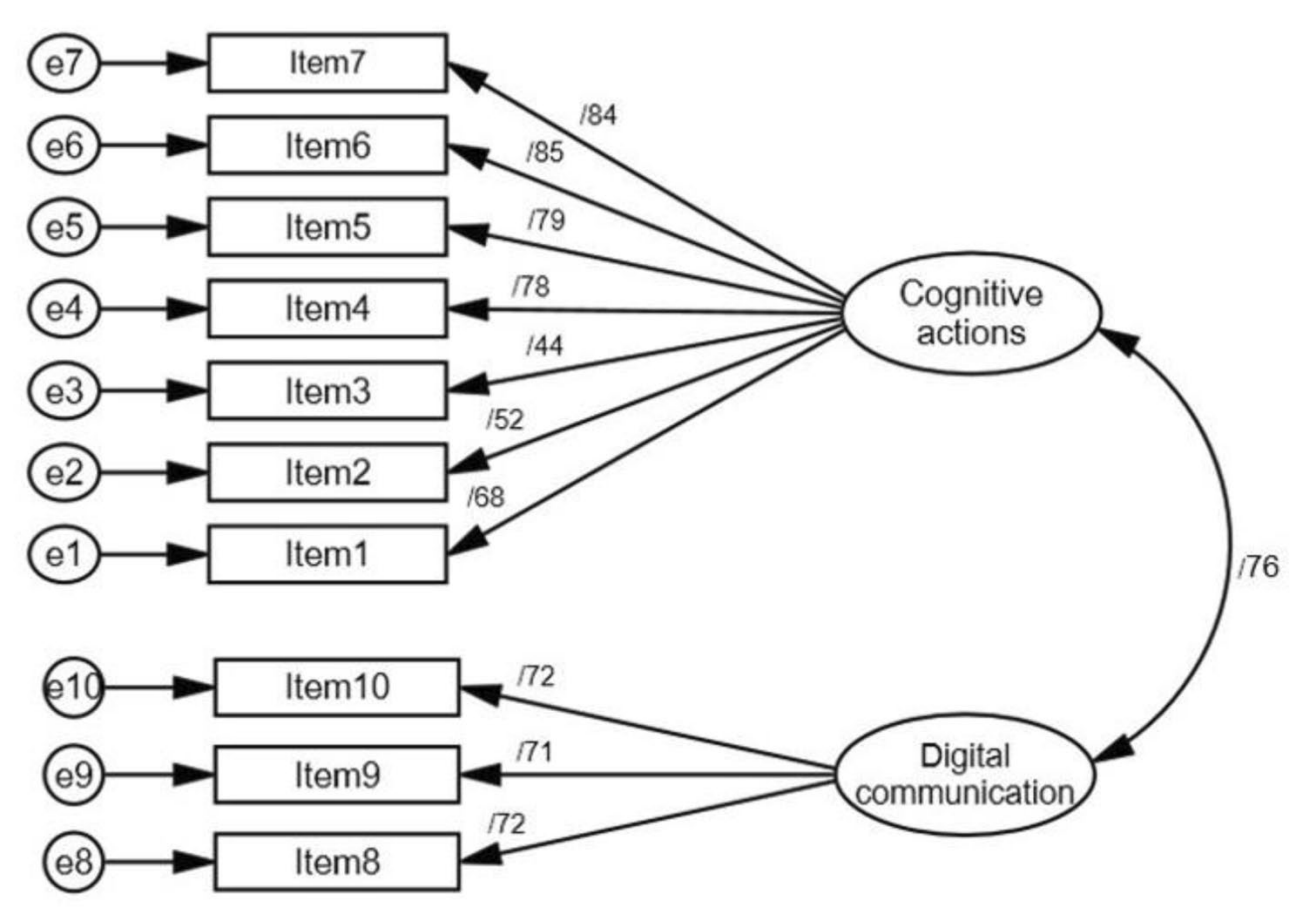
Factor structure with 10 items and two factors

Results of the CFA supported the hypothesized two-factor model with 10 items (Fig. 2). All of the items were significant in their hypothesized factor (*P*-values < 0.001), and standardized factor loadings ranged from 0.44 to 0.86. Model fit indices demonstrated acceptable model fit (normed chi-square = 2.22, RMSEA = 0.07, SRMR = 0.05, CFI = 0.96, GFI = 0.93). The correlation between factor 1 and 2 was 0.76. Moreover, HTMT was 0.68 (the criterion value was < 0.85), hence satisfying that the discriminant structure of the two factors.

The results of the hypothesis testing further supported the construct validity of Persian CeHLS-D. As hypothesized, there was a significant and strong relationship between Persian CeHLS-D and diabetes health literacy. Spearman correlation analysis revealed a moderate positive correlation between the Persian CeHLS-D mean score and patients’ perceived usefulness and importance of using the internet to find health information. As predicted, the Persian CeHLS-D score was positively associated with patients’ perceived health status and well-being and negatively associated with internet anxiety (Table 3).

**Table 3:** Correlations between persian CeHLS-D score, diabetes health literacy, and other variables

Variables	Persian CeHLS-D	
	*r*	*p*-value
Diabetes health literacy	0.664	< 0.001
Importance of using the internet	0.402	< 0.001
Usefulness of using the internet	0.304	< 0.001
Perceived health status	0.237	0.001
Well-being	0.263	< 0.001
Internet anxiety	-0.194	0.006

One-way ANOVA revealed statistically significant differences in the mean scores of the Persian CeHLS-D among the five response groups of internet use frequency (F = 8.58, *P* < 0.001). A post-hoc test for group comparisons found that the mean scores on the Persian CeHLS-D of the almost every day use group were significantly higher than the other groups. Moreover, as expected, there were statistically significant differences in the mean scores of the Persian CeHLS-D among the participants with different attitudes towards providing online healthcare services (F = 4.10, *P* = 0.018). A post-hoc test for group comparisons found that the mean scores on the Persian CeHLS-D of the participants who had a positive attitude were significantly higher than the participants who had a negative attitude. Finally, respondents who reported the pandemic increased their internet use had higher mean scores on the Persian CeHLS-D compared to those who reported the pandemic did not change their internet use (Table 4). These findings support that the Persian CeHLS-D had acceptable known-groups validity.

**Table 4:** Known-groups analysis of the persian CeHLS-D by frequency of internet use, and attitude towards online services

Group	Mean (SD)	F	*P*-value	Post-hoc test
				(Tukey-Kramer)
Frequency of internet use		8.58	< 0.001	a > b, c, d, e
Almost every day^a^	2.17 (0.76)			
Several days a week^b^	1.73 (0.79)			
Around one day a week^c^	1.57 (0.64)			
Less than one day a week^d^	1.62 (0.59)			
Almost never^e^	1.13 (0.75)			
Attitude towards online services		4.1	0.018	
Positive attitude (strongly agree or agree)^a^	1.88 (0.77)			a > c
Neutral attitude (neither agree nor disagree)^b^	1.50 (1.1)			
Negative attitude (disagree or strongly disagree)^c^	0.85 (1.1)			
Pandemic effect on internet use			0.009	
Yes, it has increased	1.99 (0.76)	-2.63		
No, nothing changed	1.69 (0.81)	(t-test)		

#### Reliability

Construct reliability results are shown in Table 2. Cronbach’s alpha, composite reliability and maximum reliability for both factors were greater than the recommended reliability threshold of 0.70. Item-total correlation coefficients ranged between 0.59 (for item three) and 0.83 (for item four) (Table 2). In the sample of 50 patients completing a second questionnaire after two weeks, the intra-class correlation coefficient was 0.91 for factor 1 and 0.86 for factor 2 (*P* < 0.001), indicating good stability over time.

## Discussion and conclusion

### Discussion

With the vast amount of health information available online, people with diabetes require strong eHealth literacy skills to effectively manage their condition. This study validated the Persian version of the CeHLS-D, a tool specifically designed to assess eHealth literacy in the context of diabetes care.

The factor analysis yielded a two-factor structure (cognitive actions for internet diabetes information, and abilities of digital communication), with acceptable factor loadings and no cross loading on the other factor. In this study, correlation between the two factors was 0.76. Although there is no single criterion that can be used to determine whether or not two constructs have discriminant validity with certainty, this value is generally considered to be evidence of discriminant validity. For example, Voorhees (2016) states that the most commonly used criterion for discriminant validity is to compare the correlation between two constructs against a fixed value of 0.85. If the correlation between two constructs is less than 0.85, then this suggests that the two constructs are sufficiently distinct to have discriminant validity [36]. Moreover, the HTMT value obtained in this study provided additional evidence to support the discriminant nature of this two-factor structure.

Internal consistency of the Persian CeHLS-D was good, with both factors above the criterion of 0.7 and confirming the reliability of the instrument. All item-total correlations were between 0.59 and 0.83, confirming good reliability. Moreover, we assessed the test-retest reliability of the Persian CeHLS-D, which was not done in the original study by Lee et al. [16]. Both factors showed good 2-week test-retest reliability estimates (0.91 and 0.86), strengthening the evidence base for the Persian CeHLS-D as a reliable tool for measuring eHealth literacy in the diabetes care.

Convergent validity was supported by confirmation of hypothesized correlations between Persian CeHLS-D and ‘diabetes health literacy’, ‘perceived usefulness and importance of the internet’, ‘perceived physical and mental health status’, and ‘internet anxiety’. These findings are, in accordance with expectations, and are supported by previous studies [16, 31, 32, 34, 37]. Another main point is that Persian CeHLS-D had the strongest relationship with diabetes health literacy (*r* = 0.66). This finding is likely due to the context-specific nature of eHealth literacy skills and the fact that patients with higher Persian CeHLS-D scores tend to be “diabetes information explorers”, able to identify good online diabetes-related information and reliable sources of this kind of information, and to resolve conflicting information [38]. This result is also consistent with findings of Lee et al.’s study of the original CeHLS-D version [16].

Known-groups analysis showed that the Persian CeHLS-D could significantly discriminate between patients who used the internet almost every day and those who used it less. The Persian CeHLS-D mean score also differed between patients whose internet use had increased during the COVID-19 pandemic compared to those whose internet use had not been affected by the pandemic. Moreover, significant differences in the Persian CeHLS-D mean scores were observed between groups of patients with different attitudes towards providing online healthcare services. These findings are supported by prior studies, which found that higher eHealth literacy is associated with frequent use of the internet, and a positive attitude towards online resources [16, 26, 27, 37].

There was a floor effect on the item “emailing”, which achieved the lowest mean score among the items. This finding suggests that a significant proportion of participants in the study may not have the ability or willingness to use email to communicate with their healthcare providers about their diabetes. This could be due to several reasons, such as, limited access to email technology, lack of familiarity with email, cultural preferences for face-to-face communication, and concerns about privacy and security [39]. In this regard, a recent study in Tehran on acceptance of Information and Communication Technology (ICT) showed that emailing was the least common ICT-based activity among people aged 55 and older [40]. Another study on patients with chronic diseases showed that patients readiness to engage in health information technology was at a medium level in Iran [41]. Considering that, the item of “emailing” might be a relatively difficult skill for the participants of this study. It is therefore recommended to revise that item to make it more accessible in future studies. Moreover, as the use of email’s capabilities plays a significant role in improving the quality of healthcare services, fostering email literacy among patients with T2DM can empower them to become active participants in their care, leading to improved health outcomes and a more patient-centered healthcare experience.

In the original Korean version of the CeHLS-D, the item of “text messaging” achieved the highest mean score among the items and showed a ceiling effect [16]. This result was almost obtained in our study as well, by having this item the highest mean score, and showing almost a ceiling effect (14.3%). This observation aligns with the widespread adoption of mobile communication in South Korea and Iran, where the mobile user rates stand about at 95% and 87% respectively. These findings suggest that text messaging has permeated daily communication and healthcare interactions in these societies, likely driven by cultural factors that promote digital engagement and technological fluency.

The current study has several methodological strengths and limitations that should be noted. The high methodological and psychometric standards applied to translate and confirm the Persian CeHLS-D content and construct validity. Test-retest reliability of the CeHLS-D was assessed and provided strong evidence that the CeHLS-D is a reliable measure of eHealth literacy in Iranian adults. However, the CeHLS-D is a self-reported measure, and does not directly measure an individual’s actual knowledge of eHealth. To address this limitation, future research could develop online versions of the Persian CeHLS-D for administration via tablets, smartphones, or email. Moreover, the study’s sample may not be representative of the broader population of diabetes patients, as those who agreed to participate may have been more interested in using the internet than other patients. This could have introduced selection bias and potentially overrepresented individuals with higher levels of eHealth literacy in the sample.

### Conclusion

This study provides initial and strong evidence that the Persian CeHLS-D has acceptable psychometric properties in patients with T2DM in Iran. Results provide evidence of acceptable reliability and construct validity as a patient-reported measure of eHealth literacy in this sample of diabetes patients.

### Electronic supplementary material

Below is the link to the electronic supplementary material.

Supplementary Material 1

## Data Availability

The data are available on reasonable request from the corresponding author.
