# Supplementary material for: Measuring electronic health literacy in the context of diabetes care: psychometric evaluation of a Persian version of the condition-specific eHealth literacy scale for diabetes
Source: BMC Med Inform Decis Mak. 2024 Jul 5;24:189. doi: 10.1186/s12911-024-02594-0 (PMC11225123; doi:10.1186/s12911-024-02594-0)
Supplement: Supplementary file 1 — Supplementary Material 1 [file 12911_2024_2594_MOESM1_ESM.docx]

**CeHLS-D questionnaire**

*These questions will help us understand your skills and abilities in using the internet to find information about managing your diabetes. Please answer each question by choosing the response that best reflects how well you are able to perform the described action at the present time.*

|  | Not at all | A little bit | Somewhat | Much | Very much |
| --- | --- | --- | --- | --- | --- |
| *When looking for information about diabetes and self-management online…*   1. To what extent are you able to think of different search words and change your search terms based on the results you found? |  |  |  |  |  |
| 1. To what extent are you able to understand the **meaning and significance** of the medical terms used in the diabetes information you found? |  |  |  |  |  |
| 1. To what extent are you able to **figure out the meaning of numeric medical examination values, like HbA1c and fasting glucose, in the diabetes information you found?** |  |  |  |  |  |
| 1. To what extent are you able to appraise the credibility of the diabetes information you found? |  |  |  |  |  |
| 1. To what extent are you able to **tell the difference between advertisements and diabetes real information on the websites you visited?** |  |  |  |  |  |
| 1. To what extent are you able to judge the trustworthiness of internet sources where you found diabetes information?" |  |  |  |  |  |
| 1. **To what extent are you able to filter applicable information that directly addressed your needs?** |  |  |  |  |  |
| 1. **To what extent are you able to** communicate with your healthcare provider about your diabetes via email? |  |  |  |  |  |
| 1. **To what extent are you able to send a text message to your healthcare provider about your diabetes status (**e.g., WhatsApp, Eitaa**)?** |  |  |  |  |  |
| 1. **To what extent are you able to share your opinions about diabetes on social media (e.g., Instagram, YouTube)?** |  |  |  |  |  |
